# LiveStories

**DOI:** 10.5195/jmla.2018.409

**Published:** 2018-04-01

**Authors:** Kelli Yakabu, MLIS Candidate, Andrea Ball

**Affiliations:** iSchool, University of Washington, Seattle, WA; Health Sciences Library, University of Washington, Seattle, WA

## OVERVIEW

LiveStories is a web-based storytelling platform that is equipped with interactive data visualization tools, drag-and-drop publishing, and its own public data library. Launched in 2013, the Seattle-based company has helped government departments and public health organizations within and outside the United States create web pages or “stories” that provide context and analysis of data [1]. Their mission is to empower users to explore data, present insights, and inspire actions through interactive stories [2]. Users are able to either upload data or use public data that are made available to them in the LiveStories data library. They can create stories for a range of audiences and communities, and the platform encourages team collaboration by allowing team members to share and comment on others’ stories.

## MAJOR FEATURES

The basic functions of LiveStories are uploading or finding data in the LiveStories data library, creating charts and stories, and publishing on the web.

Users can upload data as a comma-separated values (CSV) file or from Google Sheets; the current maximum CSV file size is about 50 MB [3]. Once data sets are uploaded, charts are automatically generated for users to view and customize. Users can choose which factors (or columns from the data set) to compare and can try out different chart types and colors. Chart types include bar charts, line charts, barbell charts, heat maps, and geographic maps. Data can be updated easily by simply uploading the new data file; even data sets that are already featured in published stories will automatically update. Help guides and videos are available to learn how to properly clean data sheets before uploading them.

Users can also find public data in the LiveStories data library. These civic data sets are pulled from sources such as the Census Bureau, including the American Community Survey, and the Centers for Disease Control and Prevention, and they are organized by subject. Health and medical care are 2 of the largest subjects, with over 700 data sets combined and growing weekly. If specific data cannot be found, users can request data be obtained and made available. The high number of health-related data sets is due to client demand. Users can search through the data library by location, source, category, or year.

**Figure 1:**
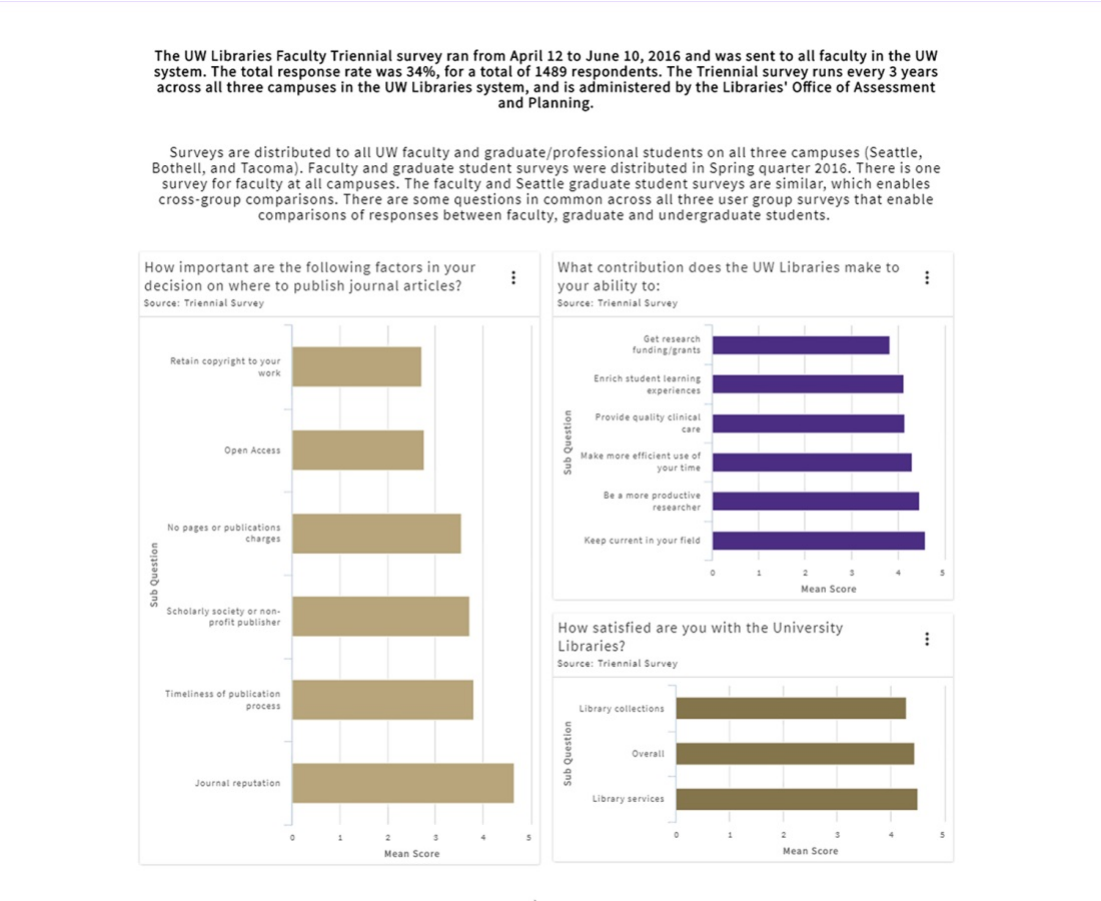
An example story created by a LiveStories client

Charts in the LiveStories data library require a set location as a benchmark to generate comparisons. These locations vary from state and city to school district and census tract. For example, if California is chosen, then the chart will show comparisons between other states and California. For all chart types except geographic maps, California will remain highlighted. Users can filter by demographic or year as well as discover comparisons between neighboring, similar in population, or specifically chosen cities, counties, and states (Figure 1). Once data are uploaded personally or found in the data library, users can “pin” charts to “dashboards” (similar to folders) for easy future access. Having the data in one place makes it easier for users to begin building stories around the data.

The story builder is easy to use with its drag-and-drop system. Modules are sections of a story that can contain charts, images, text, and even Esri maps to both present and provide context for data. These modules can be rearranged to create a desired layout. Charts include metadata information that can be visible or hidden. Example client stories, story templates, help guides, and videos are available to assist users. Users can also create stories from scratch or by modifying available example stories that are provided by LiveStories. Stories can be published to the web and shared by a hyperlink or through Facebook and Twitter, and stories can be edited and republished multiple times. Stories can be downloaded as portable document format (PDF) files as well. Individual charts can be downloaded or shared through Facebook and Twitter.

LiveStories also encourages team collaboration. Users can be on multiple teams, depending on projects, departments, or other factors. The home page features a “feed” that allows users to view and comment on edited stories, uploaded data sets, or entire dashboards. Users can also edit stories together and invite other teammates to view and provide feedback on ongoing projects. Rather than navigating email chains between colleagues, all collaboration takes place on the web-based platform, and users are notified when comments are made.

## USABILITY

LiveStories requires no coding or web design expertise, allowing users to create well-designed web pages without having to spend additional time learning special technical skills. Users are able to easily create interactive and visually appealing stories after online training that LiveStories staff provides.

Multiple data sets can be uploaded at once, and users can toggle between different chart options with a click of the mouse. The story builder is straightforward, with a drag-and-drop system. Templates, help guides, and videos are also available for a variety of topics, including how to use story design tools, store projects in a work space, and build charts. A librarian or staff member could easily maintain a published story without knowing how to code. LiveStories requires only an Internet connection and does not require users to install software.

As of November 2017, the mobile version is still in the early stages of development and is not yet fully functional.

## AUDIENCES

While LiveStories has primarily worked with city and county public health, safety, and educational departments, librarians and instructors will also find the platform useful for presenting information and collaborating on projects. LiveStories is a convenient tool for presenting information to the public in an approachable way. For example, librarians can translate PDF reports into stories, providing the main points in an easy-to-follow and interactive manner, while also linking to full reports for audiences who desire more information. Instructors can introduce LiveStories to students who are working on group projects, helping them learn more about multimodal web composition, collaborative teamwork, and design and presentation of web pages and charts for various audiences. Students conducting research can present findings concisely to help readers understand the content and main findings.

## ISSUES

The most prominent issue with LiveStories concerns sources and citations. Currently, LiveStories data sets are accompanied only by hyperlinks to the location from which the data were harvested. Depending on the source, the hyperlink could direct users to (1) the exact web page from which the data came, (2) the website home page, or (3) a page that is no longer active. There is also no indication of when the data were pulled from the website or how often the data are updated. Another issue is that the LiveStories data library only has data sets from the United States and very few from before 2000. Neither of these issues are a problem for users who are using data that they have uploaded, and LiveStories can work with and visualize international data.

## SIMILAR PRODUCTS

LiveStories is the only product on the market that offers storytelling with data visualization tools. Products like Tableau have more complex data visualization capabilities but do not allow users to create context-rich and engaging stories to accompany data. Furthermore, LiveStories’ emphasis on collaboration allows multiple users to view and edit stories, data sets, and more, while products like Tableau do not. Though data warehouses like Data-Planet have more data sets and information, they also lack the ability to present data through interactive stories.

## CONCLUSION

LiveStories provides users with the tools to easily create and publish interactive data stories that are suitable for a variety of libraries and health-related researchers. No technical expertise is required, and LiveStories provides guides, videos, and customer support to aid users who need extra help with cleaning and uploading data sets and designing stories. Readers can request a free demo with a walk-through from a LiveStories staff member and find inspiration from and view health stories that clients have built.
